# Tissue-engineered blood vessel mimics in complex geometries for intravascular device testing

**DOI:** 10.1371/journal.pone.0217709

**Published:** 2019-06-26

**Authors:** Robert Dalton Chavez, Sara Leifer Walls, Kristen O’Halloran Cardinal

**Affiliations:** Department of Biomedical Engineering, California Polytechnic State University, San Luis Obispo, California, United States of America; CVPath Institute Inc., University of Maryland, UNITED STATES

## Abstract

**Objective:**

Intravascular stents are commonly used to treat occluded arteries during coronary heart disease. After coronary stent implantation, endothelial cells grow over the stent, which is referred to as re-endothelialization. Re-endothelialization prevents blood from clotting on the stent surface and is a good predictor of stent success. Blood vessel mimics (BVMs) are *in vitro* tissue-engineered models of human blood vessels that may be used to preclinically test stents for re-endothelialization. BVMs have been developed in straight geometries. However, the United States Food and Drug Administration recommends that devices intended to treat coronary occlusions be preclinically tested in bent and bifurcated vessels due to the complex geometries of native coronary arteries. The main objectives of this study were to develop and characterize BVMs in complex geometries.

**Design:**

Bioreactors were designed and constructed so that BVMs could be cultivated in bent (>45°) and bifurcated geometries. Human umbilical vein endothelial cells were sodded onto complex-shaped scaffolds, and the resulting BVMs were characterized for cell deposition. For a final proof of concept, a coronary stent was deployed in a severely angulated BVM.

**Results:**

The new bioreactors were easy to use and mounting scaffolds in complex geometries in the bioreactors was successful. After sodding scaffolds with cells, there were no statistically significant differences between the cell densities along the length of the BVMs, on the top and bottom halves of the BVMs, or on the inner and outer halves of the BVMs. This suggests cells deposited evenly throughout the scaffolds, resulting in consistent complex-geometry BVMs. Also, a coronary stent was successfully deployed in a severely angulated BVM.

**Conclusions:**

Bioreactors can be constructed for housing complex-shaped vessels. BVMs can be developed in the complex geometries observed in native coronary arteries with endothelial cells evenly dispersed throughout BVM lumens.

## Introduction

Coronary heart disease (CHD), which is the leading cause of death in the United States [1], occurs when plaque occludes coronary arteries. Coronary occlusions can be treated with stents [2]. Stents are latticed tubes that can be crimped onto catheters and deployed at blockage sites [3]. During stent deployment, stents denude endothelial cells from vessel walls, but eventually a new endothelial lining grows over the stented region [4,5]. This re-growth is known as re-endothelialization and is important for successful healing after stent implantation. A confluent monolayer of endothelial cells modulates local hemostasis and thrombolysis and protects vascular smooth muscle cells from circulating growth-promoting factors [5].

Due to the importance of re-endothelialization, we previously developed an *in vitro* testing system that could assess new coronary stents for their re-endothelialization capacity [6–8]. The system consists of tissue-engineered blood vessels that have diameters similar to coronary arteries. We refer to the *in vitro* vessels as “blood vessel mimics” (BVMs), and they consist of a polymer scaffold with a cellular lining made of human endothelial cells and sometimes smooth muscle cells. We have deployed stents in these vessels, and the vessels have successfully exhibited re-endothelialization [6,7]. These systems are intended to reduce the number of stent configurations that proceed to animal testing by screening out stents during *in vitro* studies based on their re-endothelialization capacity. Such an approach could reduce the amount of time and resources spent on animal testing and accelerate development of coronary stents and other intravascular devices [9].

So far, BVMs have been developed and used in straight geometries, which do not mimic the bends and bifurcations observed in native coronary arteries. The United States Food and Drug Administration (FDA) recommends that *in vitro* vessels designed for engineering tests of coronary stents should simulate worst-case bends observed in native coronary arteries [10]. The FDA also recommends that stents intended for use in bifurcation lesions should be tested in mock vessels with bifurcation angles representative of the most challenging anatomies observed clinically [10]. One reason for these recommendations is that bends and bifurcations alter stent loading conditions in ways that may affect non-clinical test results [10].

In addition to affecting stent loading conditions, coronary bends and bifurcations affect re-endothelialization after stent implantation, leading to multiple pathologic events [11–16]. For example, when a stent is deployed in a coronary bend, the rigid stent may partially straighten the bend [13]. This straightening alters blood velocity profiles at the bend and reduces shear stress on vessel walls [15,16]. In regions of low shear stress, the endothelium re-establishes itself more slowly [12]. Large areas that remain devoid of endothelium are associated with intimal thickening [11], which is the cause of in-stent restenosis. Accordingly, coronary bends that have been straightened with stents are associated with major adverse clinical events [13]. Also, when a stent is deployed in a coronary bifurcation, stent struts may remain suspended over coronary ostia at the branch site [14]. The suspended stent struts may not re-endothelialize [14]. As a result, stented coronary bifurcations are associated with late stent thrombosis [14]. For these reasons, and to better align with FDA recommendations, BVMs could be developed in bent and bifurcated geometries. Testing coronary stents in bent and bifurcated BVMs may produce more clinically relevant re-endothelialization data compared to using BVMs in straight geometries.

The overall goal of the present study was to establish preliminary methods for the generation of BVMs in bent and bifurcated geometries. Achieving this goal first required developing and implementing appropriate bioreactor systems and scaffold geometries and determining whether cell deposition would be possible in these geometries. The specific aims of the study were (1) to design and construct bioreactors that could house BVMs in bent and bifurcated geometries, (2) to add scaffolds and cells into the bioreactors to generate and evaluate cell deposition in BVMs in complex geometries, and (3) to deploy a coronary stent in a complex-shaped BVM as a proof of concept.

## Materials and methods

### Bioreactor design and construction

Bioreactors were designed using SolidWorks (Waltham, MA). Prototypes were constructed based on the designs. To help ensure ease of use, off-the-shelf components were incorporated into as many aspects of the bioreactor designs and prototypes as possible. For example, the main chamber of the bioreactor consisted of a polypropylene container with a snap-on lid (Lock and Lock, Seoul, South Korea, catalog # HPL806) and easy-to-use plastic fittings (Value Plastics, Loveland, CO). Silicone tubing (Tygon, Beaverton, MI, catalog # WU-95702-06) was used to create a network of tubing to direct fluid flow. The biocompatibility of various bioreactor components has been tested by the vendors. Specifically, the polypropylene biochamber passed FDA hygiene and safety tests that assess interaction between raw materials and the human body [17], and the silicone tubing underwent USP Plastics Class VI and ISO10993 biocompatibility tests [18]. To attach the tubing to the main chamber, holes were drilled into the chamber using a handheld power drill and a 15/64” drill bit. Initially, separate bioreactors were designed and constructed for each BVM geometry. Ultimately, multifunctional bioreactors were designed and constructed to support multiple BVM geometries. More detailed parts lists and assembly instructions are available [19,20].

### Cell culture

HUVECs (Lonza, Basel, Switzerland, catalog # C2519A) were expanded in T75 and T225 flasks using 1:2 and 1:3 split ratios. Cells were cultured in EGM-2 BulletKit medium (Lonza, Basel, Switzerland, catalog # CC-4176) and maintained at 37°C and 5% CO_2_. Cells were used at passage numbers of P8 or less.

### Scaffold preparation

All scaffolds were made of tubular expanded polytetrafluoroethylene (ePTFE) (Impra Bard, Tempe, AZ). The scaffolds had an inner diameter of 4 mm. ePTFE has large nodes that are connected with small fibers. The internodal distance is considered the measure of porosity [21]. We used scaffolds with a porosity of 30 μm, an interfiber distance of approximately 3.2 μm, and a fiber width of approximately 0.2 μm. S-, L-, and U-shaped scaffolds were constructed by bending straight pieces of ePTFE into the respective shapes. S-shaped scaffolds were 7 cm long, L-shaped scaffolds were 5 cm long, and U-shaped scaffolds were 5.5 cm long. The scaffolds were secured onto female luer threaded barbed fittings using sutures. Scaffolds were sterilized via autoclave. Scaffolds were then submerged in 70% ethanol, followed by 100% ethanol for denucleation of scaffold pores [22], followed by conditioning medium consisting of M199, 15% fetal bovine serum, 1% penicillin-streptomycin, and 0.1% Fungizone. Scaffolds were left in the conditioning medium overnight at 37°C and 5% CO_2_.

### Complex-shaped BVM setup and cultivation

Multifunctional bioreactors, tubing, and other components were sterilized via ethylene oxide gas. Bioreactors were filled with bioreactor medium, which consisted of M199, 10% fetal bovine serum, 1% L-glutamine, 1% penicillin-streptomycin, 0.1% Fungizone, and 0.5% HEPES buffer. Conditioned scaffolds were placed in the bioreactors, submerged in the bioreactor medium, and secured into the desired geometry. The American College of Cardiology and the American Heart Association (ACC/AHA) classify coronary arteries as “non-angulated” if they bend less than 45 degrees, “moderately angulated” if they bend 45 to 90 degrees, and “extremely angulated” if they bend more than 90 degrees [23]. For the BVM cultivation in this study, scaffolds were secured into an L-shape to model moderately angulated coronary arteries and a U-shape to model extremely angulated coronary arteries. Any unused inlets or outlets were closed using luer caps. Bioreactor tubing was connected to peristaltic pumps, and the pumps circulated medium luminally (10 rpm, 1 mL/min) for 1 minute to remove any remaining air bubbles from the bioreactor systems. Luminal outlets were then closed, and medium was circulated transmurally (150 rpm, approximately 10 mL/min) for 10 minutes to ensure medium could move through the scaffold pores. Approximately 1.5 million HUVECs/cm^2^ were pressure-sodded onto each scaffold by injecting a HUVEC suspension transmurally as described previously [6]. Pumps circulated medium transmurally (10 rpm, 1 mL/min) for 30 minutes to help cells securely deposit onto the luminal surface of the scaffolds. Luminal outlets were then opened, and pumps circulated medium luminally (15 rpm, 1.2 mL/min) for 1 day. We previously showed that the pump setting of 15 rpm corresponds to an average shear stress of approximately 0.029 dyn/cm^2^ for both the L- and the U-shaped scaffolds [24]. All BVMs in the present study were cultivated for this 1-day period except the stented BVM and its control, which is described as follows.

### Coronary stent implantation

A bare metal stent (4 mm inner diameter x 38 mm length coronary stent, Guidant Corporation, Indianapolis, IN) was deployed into a U-shaped BVM after 14 days of cultivation. Specifically, the stent was deployed by inserting a stent-loaded balloon catheter into the luminal inlet of the multifunctional bioreactor, inflating the balloon to 8 atm for 10 seconds, deflating the balloon, and removing the catheter to leave behind the deployed stent. This stenting procedure was performed aseptically in a biological safety cabinet, then the BVM was returned to the incubator and pump. Luminal flow was maintained for 3 days after stent deployment, then the BVM was harvested for evaluation. An un-stented control was created and cultivated for comparison.

### BVM harvest and fixation

BVMs were harvested from bioreactors at the time points described in the preceding two paragraphs and were cut into 5 equal segments along their length in order to capture any differences along the length of the various geometries. The segments were cut in half so that the inner and outer curves of the segments could be analyzed separately. On each half-segment, the top and bottom edges were identified. Samples were fixed in Histochoice overnight then used for either histology, scanning electron microscopy, or fluorescence microscopy, all of which are described as follows.

### Histology

The BVMs that were cultivated for 1 day were analyzed histologically for cell deposition. After BVM harvest and fixation as described above, samples were processed and embedded in paraffin. Sections were cut at a thickness of 6 μm and mounted on slides. Sections were stained with hematoxylin and eosin (H&E) for visualization of general histological characteristics.

### Scanning electron microscopy

A subset of the BVMs that were cultivated for 1 day were analyzed via scanning electron microscopy (SEM). After BVM harvest and fixation, samples were dehydrated in a series of progressively more concentrated ethanol solutions and desiccated overnight. SEM images were acquired using a Tabletop Scanning Electron Microscope (Hitachi, Tokyo, Japan, model # TM-1000).

### Fluorescence microscopy

The BVMs that were cultivated for 1 day were analyzed via fluorescence microscopy in order to capture images that would allow for cell counting and quantification of cell deposition. After BVM harvest and fixation, samples were stained with a nuclear-specific bisbenzimide (BBI) agent. Fluorescent images were acquired on the inner and outer halves and the top and bottom regions of all 5 segments. Fluorescence microscopy was also used to analyze the BVM cellular lining after stent deployment and in the corresponding un-stented control BVM. A BX41 microscope (Olympus, Center Valley, PA) was used to acquire the images. The images were imported into PowerPoint (version 14.4), and five white boxes were overlaid on the images. An observer blinded to the identity of the samples manually counted cells in the boxes in each image.

### Statistical methods

Microsoft Excel (version 14.4.0) was used to perform an Anderson–Darling test for normality. All data was normally distributed. Microsoft Excel was used to perform an f-test to assess whether variances between groups were equal. The variances between the inner and outer groups were equal, the variances between the top and bottom groups were equal, and the variances between the 5 regions along the length of the scaffolds were equal. Next, Microsoft Excel was used to perform a Student’s t-test (two-tailed, unpaired) for equal variance to compare inner versus outer cell densities and top versus bottom cell densities. GraphPad Prism (version 6.0f) was used to perform an ANOVA to compare cell densities in the 5 regions along the length of the BVMs. For all statistical analyses, a p-value of less than 0.05 was considered statistically significant. Microsoft Excel was used to calculate means and standard deviations. All graphs were made using GraphPad Prism.

## Results

### Design and construction of bioreactors and scaffolds

**Fig 1:**
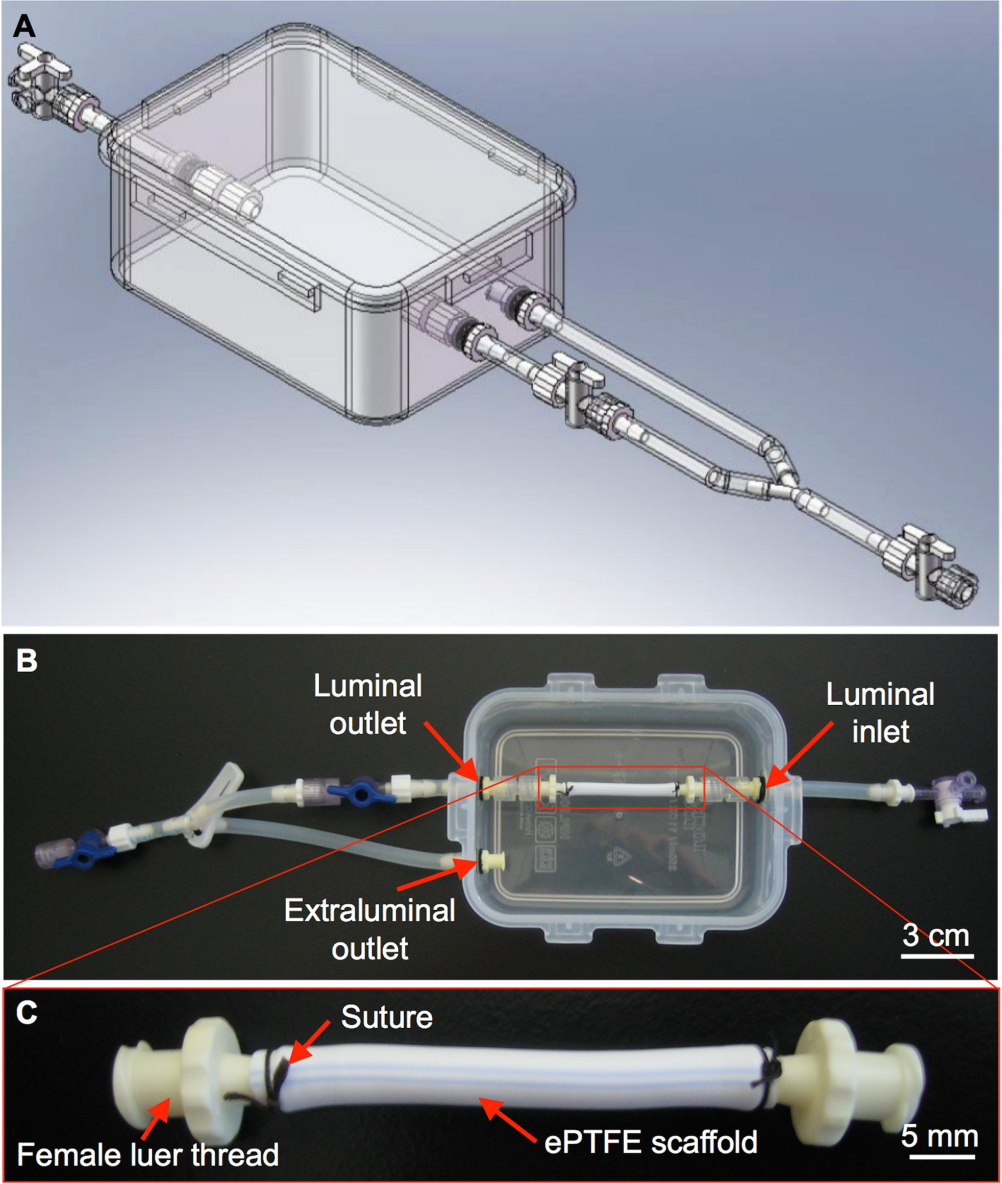
Basic bioreactor design and prototype for straight BVMs. SolidWorks was used to design a bioreactor that could house straight BVMs. (A) The main chamber of the bioreactor consisted of an off-the-shelf polypropylene container with a snap-on sealable lid. (B) Prototypes were constructed based on the design; in B, the container is shown without the lid. The bioreactor has luminal inlets and outlets to allow culture media to flow into and out of the scaffold lumen, respectively. (C) ePTFE scaffolds were mounted onto female luer threads using 2.0 silk suture and secured into the bioreactor.

BVMs were previously developed, and used for stent testing, in straight geometries [6,7]. However, the bioreactors that were used previously to cultivate straight BVMs had multiple features that could be improved, especially when increasing geometric complexity of the scaffold was desired. For example, construction of the bioreactors involved intensive machining steps. Also, operation of the bioreactors involved relatively cumbersome steps to seal media chambers with a series of screws while attempting to maintain aseptic conditions. In the present study, we went through a three-stage design process. In the first stage, BVM bioreactors were redesigned using off-the-shelf components that made them easily fabricated (Fig 1). Such components eliminated intensive machining steps and cumbersome steps to seal media chambers, which made the systems easy to reconfigure for complex geometries and easy to use.

**Fig 2:**
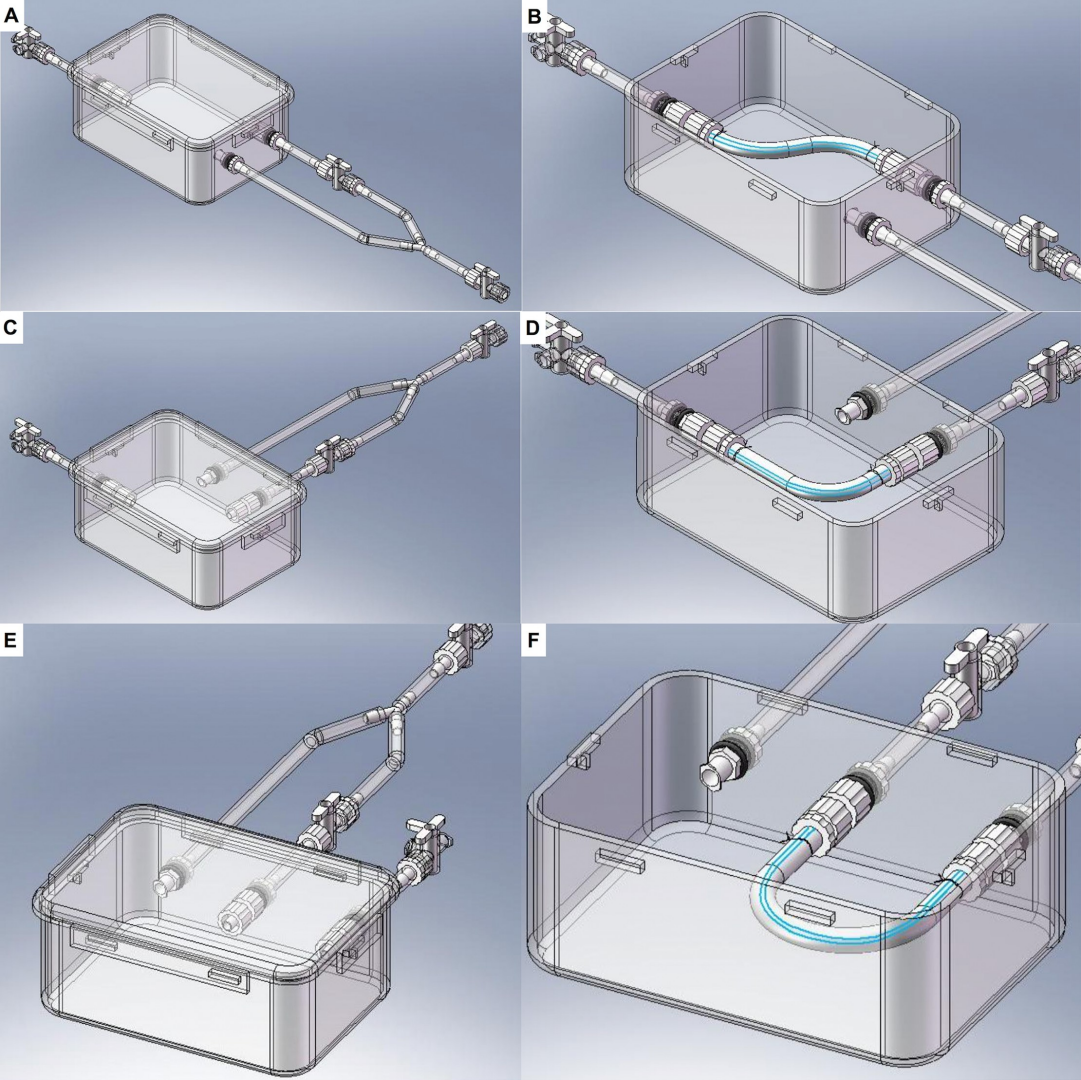
Bioreactor designs for complex-shaped BVMs. SolidWorks was used to design bioreactors that could house BVMs in some of the complex geometries of native coronary arteries as defined by the ACC/AHA. The main chamber of each new bioreactor consisted of the same easy-to-use polypropylene container as described in Fig 1. However, inlets and outlets were repositioned so that scaffolds could be mounted in the various bent geometries, resulting in three different systems to accommodate S- (A–B), L- (C–D), and U-shaped (E–F) configurations.

In the second stage of our design process, the new BVM bioreactors were further modified so that BVMs could be cultivated in the complex geometries of native human coronary arteries. Specifically, a bioreactor was designed to house an S-shaped scaffold to model a non-angulated coronary artery, another bioreactor was designed to house an L-shaped scaffold to model a moderately angulated coronary artery, and a third bioreactor was designed to house a U-shaped scaffold to model an extremely angulated coronary artery (Fig 2). Since worst-case coronary bends have been shown to exhibit a 15-mm radius of curvature [10,25], the bioreactor for U-shaped BVMs was designed so that a U-shaped scaffold could be mounted with a 15-mm radius of curvature.

**Fig 3:**
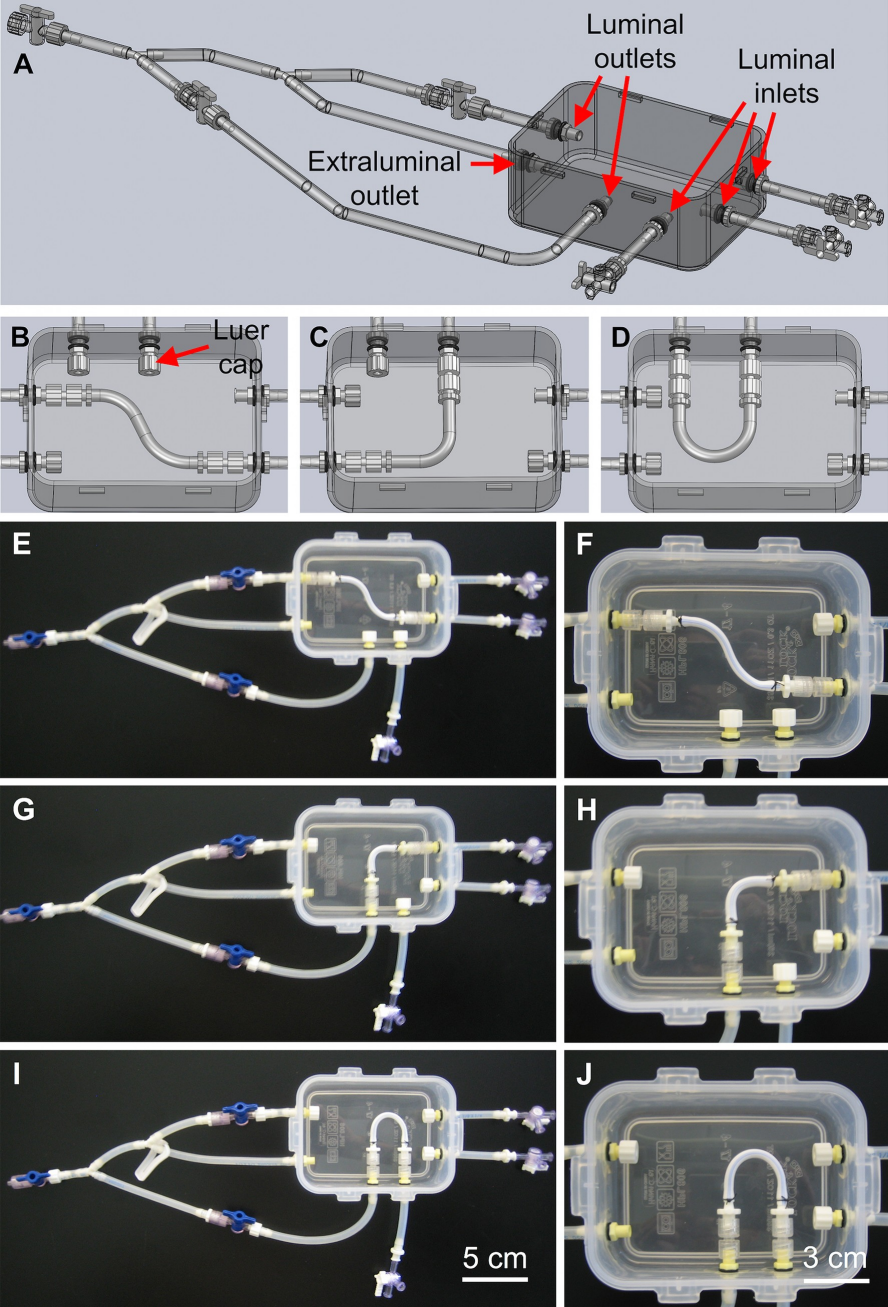
Multifunctional bioreactor design and prototype. (A–D) SolidWorks was used to design a multifunctional bioreactor that could house BVMs in a range of complex-shaped geometries. Prototypes were constructed based on the design. The same polypropylene container that was used for the straight-BVM bioreactor was used for the multifunctional bioreactor. ePTFE scaffolds were secured into the multifunctional bioreactors in S- (E–F), L- (G–H), and U-shapes (I–J). Luer caps were used to close any unused inlets or outlets.

The third stage of our design process focused on optimizing efficiency and utility of the bioreactor systems. To minimize the number of bioreactors that would need to be constructed and used on a recurring basis, the bioreactor designs for S-, L-, and U-shaped BVMs were combined into a single multifunctional bioreactor design. In this design, the single bioreactor could house any of the three scaffold geometries (Fig 3A–3D). Prototypes of the multifunctional bioreactors were constructed, and the ePTFE scaffolds that were used for straight BVMs were simply cut to appropriate lengths, bent into S-, L- and U-shapes, and mounted into the multifunctional bioreactors (Fig 3E–3J).

### Establishment of complex-shaped BVMs

**Fig 4:**
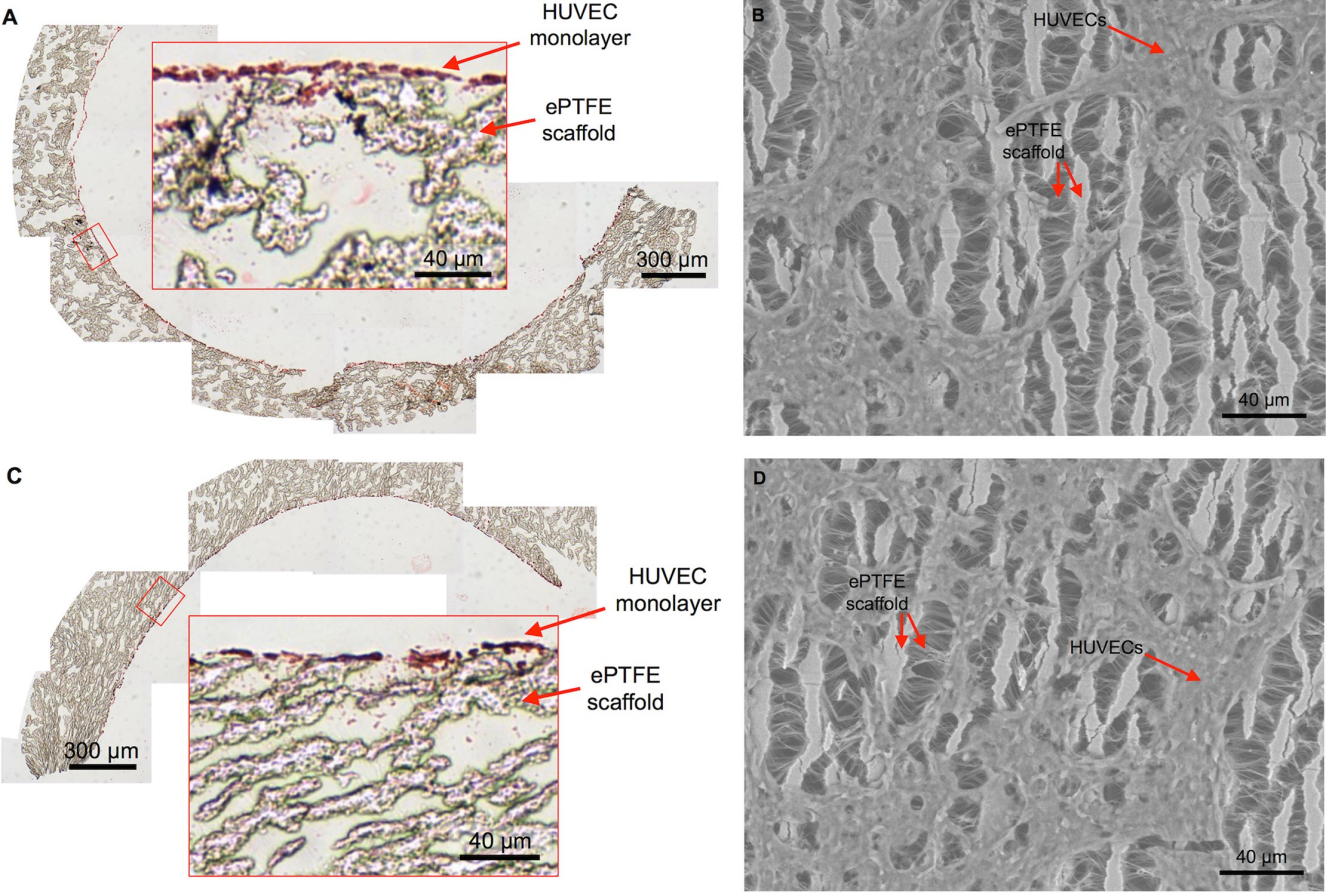
A monolayer of cells lines the luminal surface of complex-shaped BVMs. After pressure-sodding HUVECs onto the luminal surface of L- and U-shaped scaffolds and cultivating the angulated BVMs for 1 day, a monolayer of cells was visible. (A–B) On the inner curve of the angulated vessels, cells were visible in cross-sectional H&E images (A) and in *en face* SEM images (B) (n = 3). (C–D) On the outer curve of the angulated BVMs, cells were also visible in cross-sectional H&E images (C) and in *en face* SEM images (D) (n = 3). The SEM images show the surface of the ePTFE scaffolds: long vertical lines are the “nodes” of the scaffold, and short horizontal lines are the “internodal fibers.” The SEM images also show the cells sprawling across the scaffold nodes and fibers. The amount of cells appeared similar on the inner and outer halves of the angulated BVMs.

We previously showed that HUVECs are a cost-effective cell type for generating BVMs in straight geometries [8]. Therefore, in the present study, HUVECs were used for generating complex-shaped BVMs. Specifically, HUVECs were pressure-sodded onto the luminal surface of L- and U-shaped scaffolds. As mentioned in the Methods, the present study focused on generating L- and U-shaped BVMs because the ACC/AHA define L- and U-shaped vessels as “angulated” due to their more severe bends compared to S-shaped vessels, which are defined as “non-angulated” due to their less severe bends. Angulated vessels better represent the geometries of native coronary arteries where stents may be deployed. H&E staining was used to assess general histological characteristics of L- and U-shaped BVMs 1 day after pressure-sodding cells onto scaffolds. The inner and outer edges of the bends in each BVM were assessed separately. The inner and outer edges qualitatively exhibited similar cell deposition in the H&E images (Fig 4A and 4C respectively) and in the SEM images (Fig 4B and 4D respectively). While there was clearly consistent deposition of cells, the cellular lining was not yet 100% confluent at this time point.

**Fig 5:**
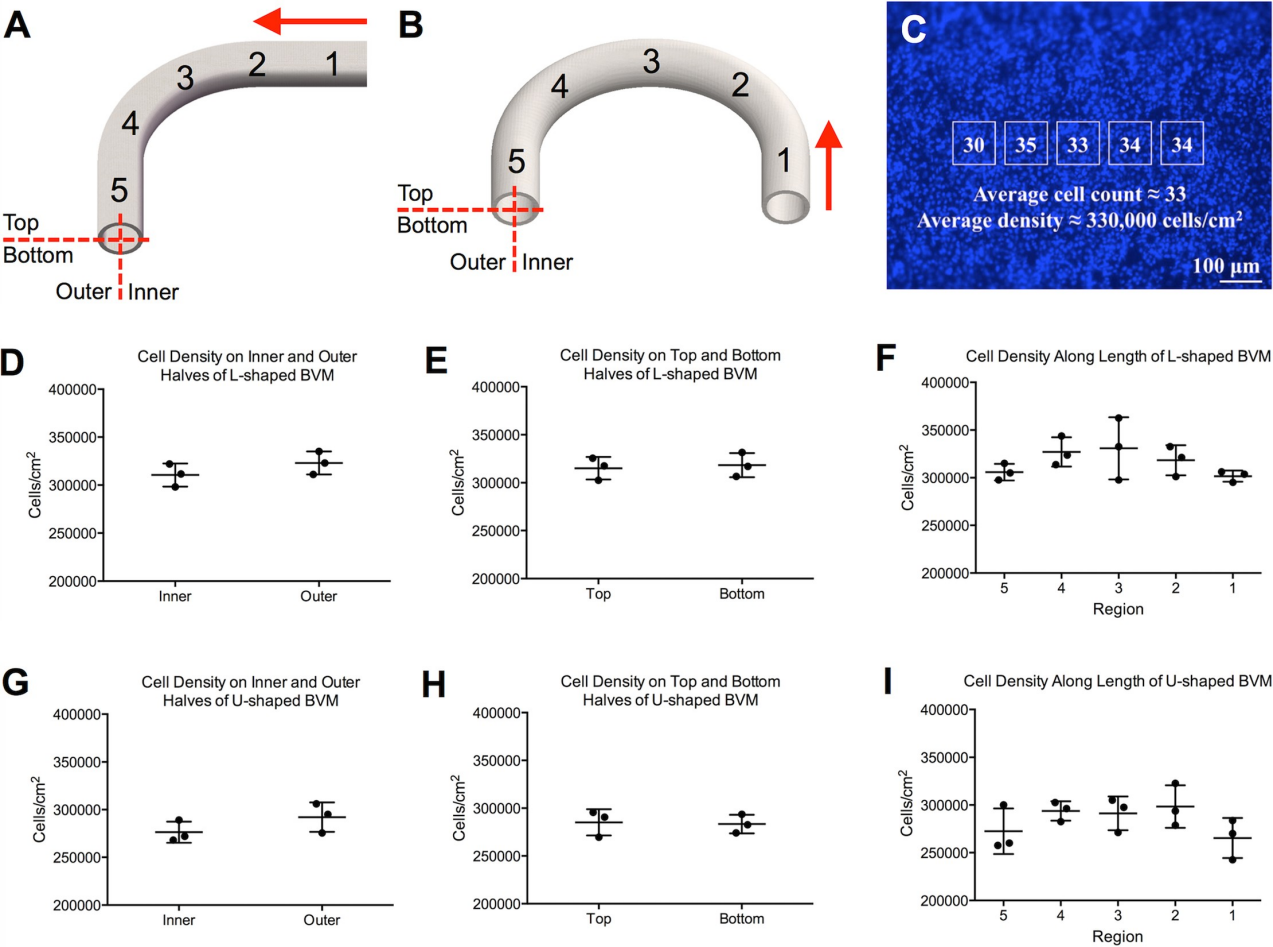
Cells deposited evenly throughout the luminal surface of L- and U-shaped scaffolds. To quantify the number of cells that deposited onto L- and U-shaped scaffolds, cells were counted in various regions throughout the luminal surface of the scaffolds after one day of cultivation. (A–B) Specifically, cells were counted on the inner and outer halves of the scaffolds, on the top and bottom halves of the scaffolds, and in five regions along the length of the scaffolds (red arrows denote the direction of fluid flow). Cell counting was performed on fluorescent BBI images. (C) Specifically, a template of five boxes was overlaid onto the BBI images, and cells in the boxes were counted and averaged to obtain a number of cells per unit area for each image. (D–F) In L-shaped BVMs, there was no statistically significant difference between the cell densities on the inner and outer halves (p = 0.272, n = 3), on the top and bottom halves (p = 0.765, n = 3), or along the length (p = 0.279, n = 3). (G–I) In U-shaped BVMs, there was no statistically significant difference between the cell densities on the inner and outer halves (p = 0.223, n = 3), on the top and bottom halves (p = 0.860, n = 3), or along the length (p = 0.244, n = 3).

Cell deposition on the angulated scaffolds was also assessed quantitatively. BVMs were stained with the nuclear-specific BBI agent and imaged via fluorescence microscopy. Cells were counted in all 5 regions along the length of the BVMs, on the inner and outer halves of the BVMs, and on the top and bottom halves of the BVMs (Fig 5A and 5B). Cell counts were converted to cell densities (Fig 5C). There were no statistically significant differences between the cell densities along the length of the BVMs, on the top and bottom halves of the BVMs, or on the inner and outer halves of the BVMs for both L- and U-shaped geometries (Fig 5D–5I). Data is provided in S1 File. These quantitative results confirmed that cells successfully deposited throughout the complex-shaped scaffolds as observed qualitatively in H&E and SEM images.

### Stent implantation and assessment

**Fig 6:**
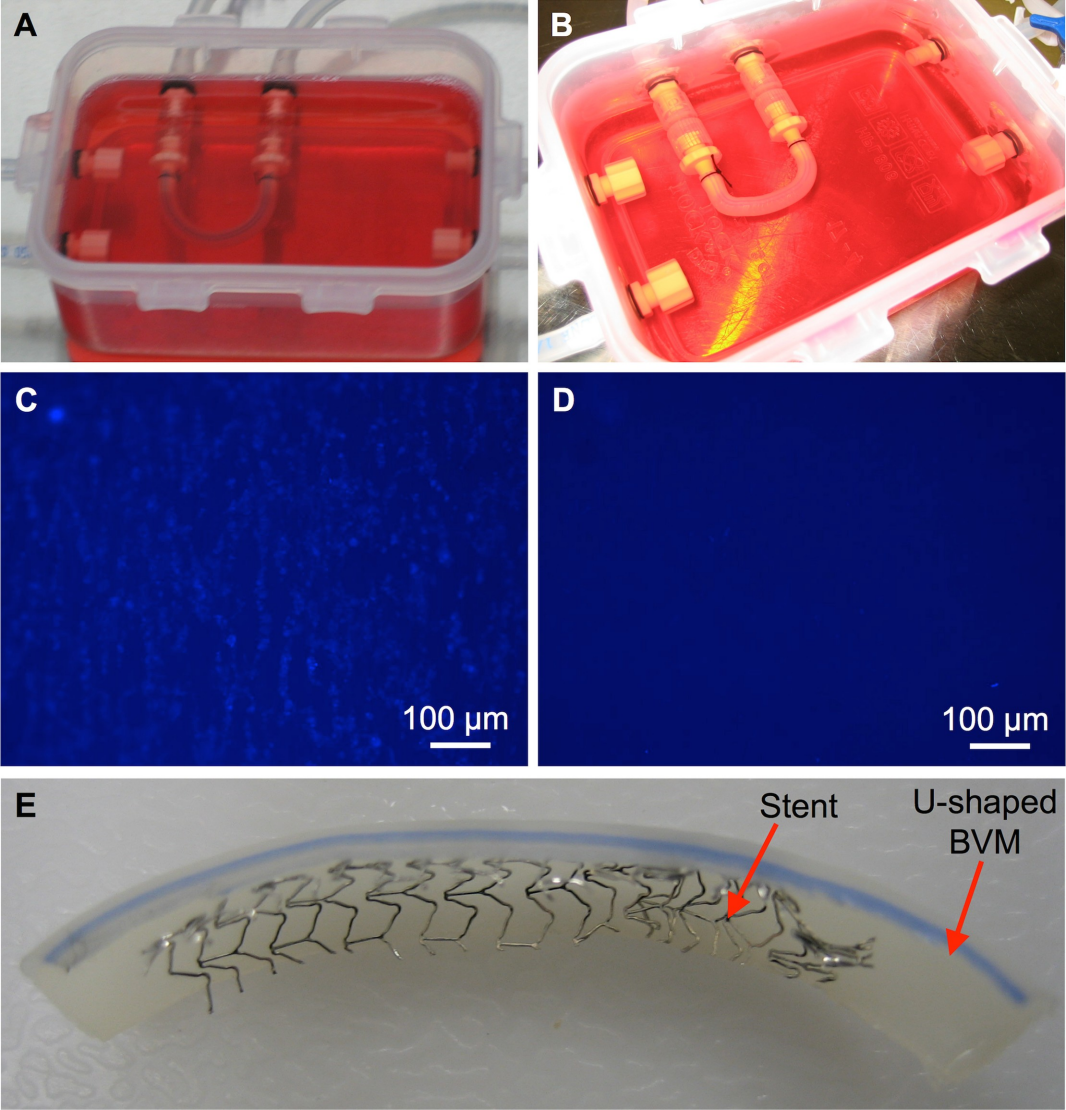
Stent deployment in a severely angulated BVM. U-shaped BVMs were cultivated for 14 days. At the 14-day time point, (A) a control U-shaped BVM was left un-stented, while (B) a coronary stent was deployed in another U-shaped BVM. The BVMs were further cultivated for three days. Finally, fluorescent BBI images of the luminal surface of the BVMs were acquired to assess the cellular lining. (C) The control BVM exhibited a cellular lining, while (D) the stented BVM did not exhibit a cellular lining in the stented region. (E) The stented BVM was cut open and it was observed that proper placement and deployment had occurred.

To test whether deployment of a coronary stent was feasible in an angulated BVM, a bare metal stent was deployed in a U-shaped BVM. Specifically, a stent-loaded catheter was introduced into the silicone tubing of a multifunctional bioreactor. The stent and catheter were visible through the silicone tubing. The catheter was pushed through the silicone tubing until reaching the U-shaped BVM that had been cultivated in the chamber. The catheter was pushed into the BVM until the stent was positioned near the center of the U-shaped vessel. The catheter and stent were both visible through the BVM walls. The stent was then deployed in the BVM. As the stent was deployed, the U-shaped BVM partially conformed to the shape of the rigid stent (Fig 6A and 6B). After further cultivation, the stented BVM along with the un-stented control were harvested and assessed via fluorescence microscopy. The un-stented control U-shaped BVM exhibited an endothelial lining (Fig 6C), while the stented U-shaped BVM exhibited almost no endothelial lining in the stented region (Fig 6D), suggesting that endothelial denudation occurred. The BVM was cut open to confirm that the stent deployed (Fig 6E), and it was observed that proper placement and deployment had occurred.

## Discussion

In the present study, we designed and constructed new BVM bioreactors with off-the-shelf, easy-to-use components, eliminating the difficult aspects of previous designs and allowing cultivation of BVMs in a range of vessel geometries. A multifunctional system was designed and fabricated, reducing the overall number of bioreactors. Closing off unused inlets or outlets with luer caps when inserting various scaffold geometries was easy. Overall, the multifunctional bioreactors streamlined development of complex-shaped BVMs.

We next added scaffolds and cells into the multifunctional bioreactors. We used scaffolds made of ePTFE. While ePTFE has been shown to exhibit an elastic modulus that is different than that of native arteries [26], ePTFE has been successfully used as a vessel replacement in humans for decades, and in particular its re-endothelialization capacity *in vivo* has been well characterized [27]. The inner diameter of the main coronary arteries in humans can range from 1.6 to 4.5 mm [28], so we used ePTFE scaffolds with an inner diameter that falls within this range, specifically a 4-mm inner diameter. The scaffolds were easily cut to appropriate lengths and bent into appropriate shapes to model coronary geometries. HUVECs were deposited throughout scaffold lumens, and there were no statistically significant differences between cell densities in any of the regions that were assessed. Because cells deposited fairly evenly on L- and U-shaped scaffolds, we hypothesize that cells would also deposit fairly evenly on less angulated scaffolds, such as S-shaped scaffolds, as well. Overall, initial setup and cell deposition within BVMs in coronary geometries was successful.

As a proof of concept for device deployment, we utilized an extremely angulated BVM for coronary stent implantation. The deployment was simple and able to be performed visually in a biological safety cabinet. Although the cellular response to stents implanted in this model will need to be further evaluated, and will be addressed in the limitations below, this proof of concept illustrated that stent deployment is not only possible but quite efficient, and it was easy to place the device in the new bioreactors and complex-shaped BVM presented in the current work.

The multifunctional bioreactor and complex-shaped BVMs in the present study are different from previous tools in multiple ways. Previous bioreactors in our laboratory and in other laboratories have been used to generate tissue-engineered blood vessels for stent testing [6,7,29], but these previous bioreactors are not capable of housing vessels in the complex geometries of coronary arteries. To mimic complex-shaped vasculature, others have created flow chambers for cultivating blood vessel models in complex geometries [30]. However, these flow chambers generate micro-scale vessels that are too small for receiving coronary stents. Our multifunctional bioreactor combines advantages of these previous tools. Specifically, our bioreactor can house vessels in complex geometries with coronary-sized lumens, making our model capable of receiving a coronary stent.

There are limitations to the present study. One limitation is the time points that were used for BVM cultivation and stent implantation. In Figs 4 and 5, BVMs were cultivated for only 1 day, and in Fig 6, a stent was implanted for only 3 days. In our previous studies on straight BVMs, vessels were cultivated for 7–14 days followed by an additional 7–14 days of stent implantation [6,7]. These longer time points may allow for more obvious changes in tissue growth to appear, making re-endothelialization assessments easier. For the present study, cell deposition at early time points was the focus. Additionally, in the present study only one cell type was used—HUVECs. Since endothelial cells form only monolayers [31], a thick vascular tissue capable of withstanding stent deployment and capable of regenerating after stent deployment may not be able to form using only HUVECs. One potential solution involves first adding smooth muscle cells to the complex-shaped scaffolds followed by endothelial cells, since smooth muscle cells have been shown to improve endothelial cell attachment to synthetic grafts [8,19,32–34]. Another potential solution involves adding circulating endothelial progenitor cells to the bioreactor system since they have been shown to accelerate re-endothelialization [35–37] or adding vascular-resident endothelial progenitor cells since current findings support their involvement in tissue regeneration processes [38]. Another limitation in the present study is that the cellular linings were not 100% confluent, due to the early time points selected, and most endothelial cells were denuded from the vessel during stent deployment, which could prevent the re-endothelialization process. These limitations could be addressed with the previously mentioned potential solutions—longer cultivation periods and different cell types, both of which may help form a thicker, confluent cellular lining. Another limitation in the present study is that the shear stresses were less than the 15+ dyn/cm^2^ shear stresses observed in native arteries [24,39]. However, our previous BVMs in straight geometries were cultivated using similar conditions, and the straight BVMs exhibited thick, confluent cellular linings [6,7]. This suggests that sub-physiologic shear stresses are adequate for *in vitro* models assessing re-endothelialization. Additional work would need to be performed to thoroughly address these limitations. While the complex-shaped BVMs in the present study were not used to comprehensively evaluate stent re-endothelialization, the goal of establishing methods for bioreactor construction, cell seeding of complex-shaped scaffolds, and proof-of-concept stent deployment in a complex-shaped BVM was achieved.

**Fig 7:**
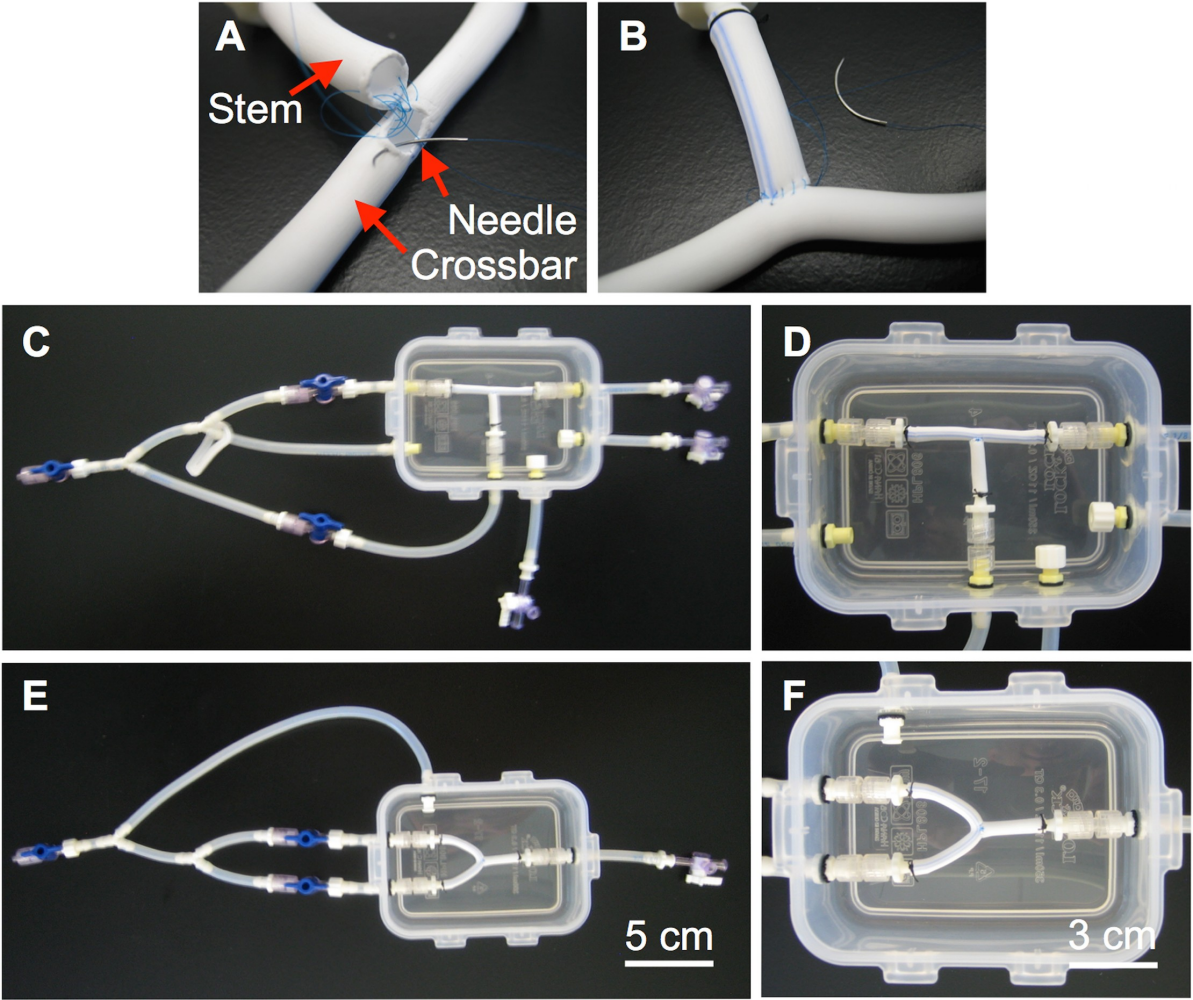
Bioreactor prototypes for bifurcated BVMs. (A–B) Bifurcated scaffolds were created by suturing together two straight scaffolds using 6.0 silk suture. (C–D) The bifurcated scaffold was secured into a T-shape in the multifunctional bioreactor. As with the angulated BVMs, luer caps were used to close unused inlets and outlets in the multifunctional bioreactor. (E–F) A separate bioreactor was constructed to house Y-shaped BVMs. Custom Y-shaped bioreactor construction was simple and straightforward using the methods presented in this paper. To create a Y-shaped scaffold, the T-shaped scaffold was simply bent into a Y-shape.

The present study could lead to a range of future studies. Immediate next steps could include longer time points or more in-depth evaluation of device implantation, as discussed above. Additionally, other geometries could be explored. For example, we have initiated development of BVMs in bifurcated geometries to model native coronary arteries with bifurcations (Fig 7). These geometries, as well as other more tortuous paths, could be developed and characterized. Also, flow characteristics in the various complex-shaped BVM geometries could be characterized [24]. Finally, other types of devices could be tested in the complex-shaped BVMs, including drug-eluting stents, balloons, and intravascular imaging modalities.

In summary, multifunctional bioreactors were designed and constructed for housing Blood Vessel Mimics in complex geometries, scaffolds and cells were added into the bioreactors to generate BVMs in complex geometries, and a coronary stent was deployed in an extremely angulated BVM as a proof of concept. While the complex-shaped BVMs presented here were not used to fully assess re-endothelialization, the outcomes from this work provide a foundation for future work that will focus on further developing and utilizing complex-shaped BVMs as *in vitro* models for early-stage preclinical use.

## Supporting information

S1 File Supporting information for Fig 5 data. (XLSX)
